# Risk factors for postoperative thrombotic complications after meningioma resection: a retrospective single-center study in China

**DOI:** 10.3389/fneur.2025.1579384

**Published:** 2025-06-02

**Authors:** Yingying Kong, Beibei Jin, Yijie Zhang, Jianhai Long

**Affiliations:** Department of Pulmonary and Critical Care Medicine, Beijing Tiantan Hospital, Capital Medical University, Beijing, China

**Keywords:** meningioma, deep vein thrombosis (DVT), venous thromboembolism (VTE), pulmonary embolism, age

## Abstract

**Objective:**

To explore the incidence and risk factors for deep vein thrombosis (DVT) and pulmonary embolism (PE) following surgical intervention for meningioma.

**Methods:**

In this retrospective, observational study, we enrolled 9,067 patients with histologically confirmed meningiomas who underwent surgical resection at our institution between January 2019 and June 2024. Demographic data (including gender, age, and geographic region) and information on comorbidity, and postoperative complications were documented and analyzed. The incidences of postoperative DVT and PE were also recorded. Risk factors for DVT and PE were identified using univariate and multivariate logistic regression analyses and restricted cubic splines.

**Results:**

Among the 9,067 patients who underwent meningioma surgery, 766 (8.4%) developed deep venous thrombosis (DVT) and 32 (0.35%) developed pulmonary embolism (PE). The mean age for patients with DVT was 59.39 ± 9.85 years, and for those with PE, it was 62.22 ± 9.86 years, both significantly higher than the overall patient population (*p* < 0.001). Geographically, the highest incidences of DVT and PE were found in Northeast, North, and East China, with provinces such as Hebei, Shandong, and Inner Mongolia reporting the highest rates. Associated risk factors for DVT included advanced age, asthma, heart failure, hypertension, and pneumonia. The associated risk factors for PE were age, DVT, pneumonia, and renal insufficiency. Multivariate analysis identified age, intracerebral hemorrhage (ICH), atrial fibrillation, heart failure, hyperlipidemia, varicose veins, hypothyroidism, hypoproteinemia, pneumonia, anemia, central nervous system infection as significant predictors for DVT, and age (OR: 1.05; 95% CI: 1.01–1.09), pneumonia (OR: 3.60; 95% CI: 1.65–7.85), and SDVT (OR: 15.88; 95% CI: 6.94–36.35) for PE. Nomogram models demonstrated strong predictive performance, with ROC values of 0.754 for DVT and 0.886 for PE.

**Conclusion:**

The incidences of DVT and PE following meningioma surgery were 8.4 and 0.35%, respectively. Age, comorbidities, and postoperative complications significantly influence the risk of these thromboembolic events. The study highlights the need for early identification and tailored prevention strategies, and the developed predictive models offer useful tools for clinical decision-making. However, the models’ overestimation of risk warrants further refinement to enhance their clinical applicability.

## Introduction

Meningiomas are common benign intracranial tumors, with a prevalence of approximately 97.5 per 100,000 (1). Although surgical resection remains the primary treatment for symptomatic cases, postoperative venous thromboembolism (VTE), including deep venous thrombosis (DVT) and pulmonary embolism (PE), is a major cause of morbidity and mortality (1–3). Studies show that postoperative VTE in patients with meningioma is significantly associated with prolonged hospitalization and elevated rates of postoperative complications, including stroke, sepsis, unanticipated intubation, hospital readmission, and in-hospital mortality (4). However, the data on VTE after meningioma surgery exhibit significant fluctuations. An early study reported an incidence of VTE as high as 72% (5), while a more recent study indicated a much lower incidence ranging from 3 to 4% (6). The incidence of DVT following meningioma surgery is reported to be around 8.71–14.10%, while PE occurs in approximately 0.7–1.9% of patients (1–3). Investigating the specific incidence and risk factors for postoperative VTE is instrumental in the prevention and management of this condition.

The exact pathophysiology of increased risk for developing VTE after meningioma resection remains unknown, but some hypotheses have included the following: Blood stasis, Brain thromboplastin proliferation, and a quantity of tumor-released hormonal and inflammatory factors (1, 7). Postoperative VTE following meningioma surgery is influenced by several well-recognized risk factors, including advanced age (8), elevated body mass index (BMI), cardiovascular conditions, and immobility after surgery (9). Understanding these factors is crucial for enhancing postoperative care and developing effective prevention strategies. However, current research continues to explore improved risk models, and there is no universally accepted preventive protocol (10). This gap highlights the need for further research to refine risk assessments and establish more effective prophylactic measures.

In the present study, we performed multivariate analyses of DVT and PE in patients who had undergone meningioma surgery, using recent, large-sample data from the Tiantan Neurosurgery Center.

## Materials and methods

### Patients

In this study, we enrolled 9,067 patients with intracranial mass lesions diagnosed via CT or MRI, who were aged ≥ 18 years, had undergone resection procedures, and were histopathologically confirmed to have meningiomas at our center between January 2019 and June 2024. Demographic data (including gender, age, and geographic region) and information on intra-and postoperative complications (e.g., intracerebral hemorrhage, DVT, and PE) were retrieved from the electronic medical records management system. This retrospective cross-sectional study included all patients who underwent surgery during the study period and followed their hospital course until discharge. All participants provided written informed consent upon admission, which also granted permission for the use of their medical data for research purposes.

The inclusion criteria were as follows: (i) complete data from the medical history, imaging studies, and perioperative records; (ii) surgical resection and a histopathological diagnosis of meningioma; (iii) radiotherapy-and/or chemotherapy-naive; (iv) age ≥ 18 years; and (v) signed informed consent for data use after admission.

Patients who were reoperated on for meningioma recurrence were excluded.

### DVT assessment

Symptomatic deep vein thrombosis (SDVT): bedside ultrasonography of both lower extremities was performed whenever a patient exhibited symptoms, such as pain, swelling, and/or pitting edema in the affected limb, or with marked changes in D-dimer concentrations. Ultrasonography was performed using the HS50 color Doppler ultrasound system (Samsung, Seoul, Korea), with the probe frequency set at 5–10 MHz. During the examination, if no blood flow signal was detected within the venous lumen, the probe was applied directly to the site of the dilated vein to observe for compression. The presence of a non-compressible or only partially compressible vein indicated a positive finding for DVT.

### PE assessment

Symptomatic pulmonary embolism (SPE): PE was assessed in accordance with the *Chinese Guidelines for the Diagnosis, Treatment and Prevention of Pulmonary Thromboembolism (2018)*. PE diagnosis was confirmed by imaging studies (computed tomography (CT) pulmonary angiography) on the basis of clinical symptoms, namely dyspnea, chest pain, syncope, and elevated D-dimer concentration. In some patients, due to limited conditions (such as renal insufficiency or unstable vital signs), a diagnosis of pulmonary embolism was made through bedside echocardiography, which reveals a free-floating thrombus in the right ventricle.

### Thrombosis prevention protocol in neurosurgery

Patients are encouraged to get out of bed and engage in activity with family assistance within 24 h post-surgery. If a patient is unable to get out of bed after 24 h and deep venous ultrasound confirms the absence of lower extremity thrombosis, intermittent pneumatic compression (IPC) is applied, with a daily duration of more than 18 h.

### Intracerebral hemorrhage assessment

Preoperatively, patients commonly present with symptoms including headaches, vomiting, elevated blood pressure, and varying degrees of altered consciousness. ICH can be identified through head CT or MRI scans. During surgery, any ICH occurring outside the surgical site is detected either through direct visualization or by using imaging techniques such as intraoperative ultrasound or CT. Postoperatively, patients undergo routine head CT scans at intervals of 2–6 h, 24 h, and 72 h following surgery, as well as whenever there is a change in the patient’s level of consciousness. ICH is confirmed via head CT scans.

### Statistical analysis

Data analysis was performed using STATA/MP software (version 18.0; StataCorp, College Station, TX, United States). Normally distributed continuous variables are presented as mean ± standard deviation, skewed continuous data are described as median (interquartile range), and categorical variables are reported as number (percentage). The chi-square test and Kruskal–Wallis rank-sum test were used to analyze differences in the distributions of DVT and PE. Univariate and multivariate logistic regression analyses and restricted cubic splines were used to examine the associations between sex, age, region, and ICH as variables associated with the occurrence of DVT and PE. *p* < 0.05 was considered statistically significant.

## Results

### Baseline, comorbidity, and complications characteristics of patients with DVT/PE

**Figure 1:**
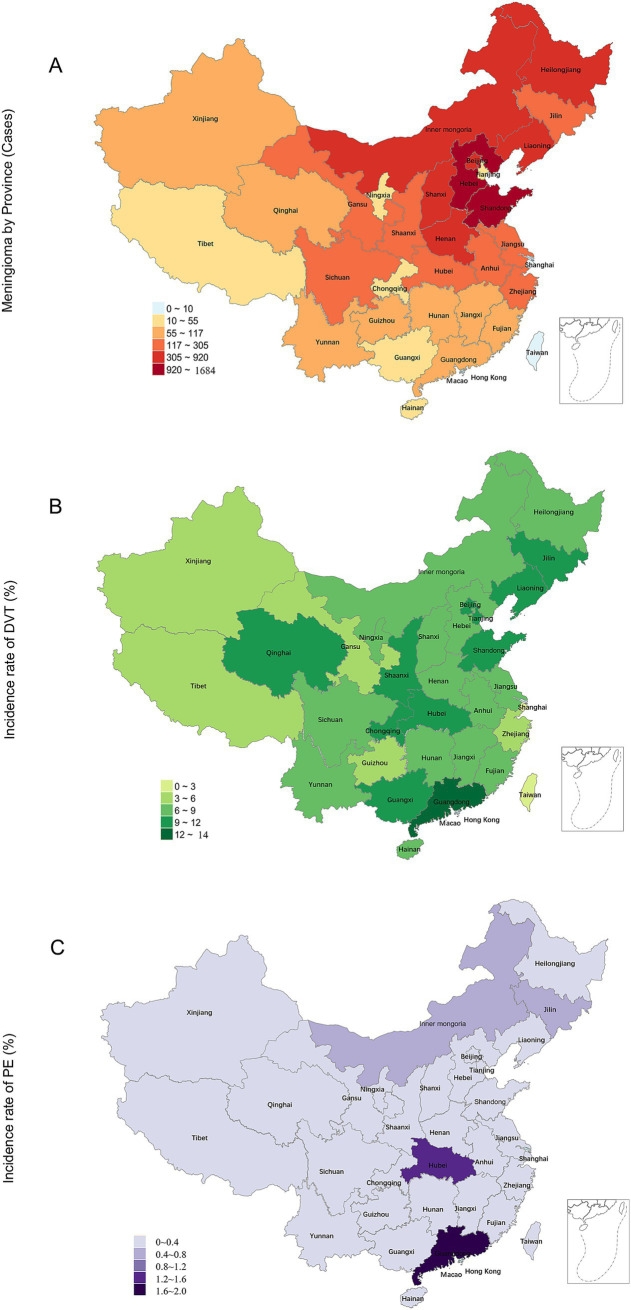
Distribution of meningioma surgeries and the incidence rates of postoperative DVT/PE in China. **(A)** Frequency distribution of meningioma surgeries; **(B)** Frequency distribution of DVT incidence (%); **(C)** Frequency distribution of PE incidence (%). DVT, deep vein thrombosis; PE, pulmonary embolism.

A total of 9,067 patients underwent surgical procedures for meningioma during the study period. Among them, 766 (8.4%) experienced SDVT and 32 (0.35%) developed SPE. Women comprised 72.10% of the total patient population (71.3% for those with SDVT and 68.8% for those with SPE). The patients’ mean age was 52.63 ± 11.19 years. Patients who developed SDVT or SPE after surgery were significantly older compared with the overall baseline population (*p* < 0.001). Specifically, the mean age was 59.39 ± 9.85 years for patients with SDVT and 62.22 ± 9.86 years for those with SPE (Table 1). Northeast China, North China, and East China were the top three regions with the highest prevalences of SDVT/SPE, and the three most affected provinces were Hebei, Shandong, and Inner Mongolia. The incidence of postoperative SDVT exceeded 10% in Guangdong, Guangxi, Qinghai, Shandong, Beijing, and Chongqing, with no statistically significant differences between these provinces. The incidence of postoperative SPE exceeded 1.0% in Guangdong and Hubei (1.72 and 1.24%, respectively), with no statistically significant differences between these provinces (Figure 1; Supplementary Table 1).

**Table 1:** Baseline, pre-and postoperative characteristics of DVT/PE patients in this study.

Item		Total *n* = 9,067	DVT		*p*	PE		*p*
			No (*n* = 8,301)	Yes (*n* = 766)		No (*n* = 9,035)	Yes (*n* = 32)	
Male, n, %		2,530 (27.9%)	2,310 (27.8%)	220 (28.7%)	0.598	2,520 (27.9%)	10 (31.2%)	0.672
Age, year, mean(SD)		52.63 (11.19)	52.01 (11.10)	59.39 (9.85)	<0.001	52.60 (11.181)	62.22 (9.86)	<0.001
Regions	Northeast	3,974 (43.8%)	3,642 (43.9%)	332 (43.3%)	0.635	3,957 (43.8%)	17 (53.1%)	0.708
	North China	1,539 (17.0%)	1,407 (17.0%)	132 (17.2%)		1,535 (17.0%)	4 (12.5%)	
	Eastern China	1,889 (20.8%)	1,714 (20.7%)	175 (22.8%)		1,883 (20.9%)	6 (18.8%)	
	Central South	892 (9.8%)	822 (9.9%)	70 (9.1%)		888 (9.8%)	4 (12.5%)	
	Northwest	308 (3.4%)	287 (3.5%)	21 (2.7%)		307 (3.4%)	1 (3.1%)	
	Southwest	461 (5.1%)	425 (5.1%)	36 (4.7%)		461 (5.1%)	0 (0.0%)	
COPD, n, %		3 (0.0%)	2 (0.0%)	1 (0.1%)	0.233	3 (0.0%)	0 (0.0%)	1.000
Asthma, n, %		24 (0.3%)	19 (0.2%)	5 (0.7%)	0.047	24 (0.3%)	0 (0.0%)	1.000
OSAHS, n, %		3 (0.0%)	2 (0.0%)	1 (0.1%)	0.233	3 (0.0%)	0 (0.0%)	1.000
Atrial fibrillation, n, %		31 (0.3%)	20 (0.2%)	11 (1.4%)	<0.001	31 (0.3%)	0 (0.0%)	1.000
Heart failure, n, %		52 (0.6%)	32 (0.4%)	20 (2.6%)	<0.001	50 (0.6%)	2 (6.2%)	0.014
CHD, n, %		70 (0.8%)	59 (0.7%)	11 (1.4%)	0.047	70 (0.8%)	0 (0.0%)	1.000
Hypertension, n, %		2,093 (23.1%)	1,836 (22.1%)	257 (33.6%)	<0.001	2,081 (23.0%)	12 (37.5%)	0.059
Hyperlipidemia, n, %		246 (2.7%)	206 (2.5%)	40 (5.2%)	<0.001	245 (2.7%)	1 (3.1%)	0.586
Varicose veins, n, %		53 (0.6%)	41 (0.5%)	12 (1.6%)	0.001	53 (0.6%)	0 (0.0%)	1.000
Diabetes, n, %		812 (9.0%)	714 (8.6%)	98 (12.8%)	<0.001	807 (8.9%)	5 (15.6%)	0.203
Hypothyroidism, n, %		212 (2.3%)	166 (2.0%)	46 (6.0%)	<0.001	212 (2.3%)	0 (0.0%)	1.000
Hyperthyroidism, n, %		30 (0.3%)	28 (0.3%)	2 (0.3%)	1.000	30 (0.3%)	0 (0.0%)	1.000
Hypopituitarism, n, %		5 (0.1%)	5 (0.1%)	0 (0.0%)	1.000	5 (0.1%)	0 (0.0%)	1.000
Cirrhosis, n, %		4 (0.0%)	3 (0.0%)	1 (0.1%)	0.298	4 (0.0%)	0 (0.0%)	1.000
Hypoproteinemia, n, %		1,131 (12.5%)	940 (11.3%)	191 (24.9%)	<0.001	1,125 (12.5%)	6 (18.8%)	0.280
Malignancy, n, %		43 (0.5%)	40 (0.5%)	3 (0.4%)	1.000	43 (0.5%)	0 (0.0%)	1.000
NS, n, %		2 (0.0%)	2 (0.0%)	0 (0.0%)	1.000	2 (0.0%)	0 (0.0%)	1.000
Renal insufficiency, n, %		46 (0.5%)	32 (0.4%)	14 (1.8%)	<0.001	44 (0.5%)	2 (6.2%)	0.011
SLE, n, %		5 (0.1%)	4 (0.0%)	1 (0.1%)	0.357	5 (0.1%)	0 (0.0%)	1.000
Pneumonia*, n, %		421 (4.6%)	288 (3.5%)	133 (17.4%)	<0.001	410 (4.5%)	11 (34.4%)	<0.001
Anemia, n, %	Normal	8,461 (93.3%)	7,813 (94.1%)	648 (84.6%)	<0.001	8,436 (93.4%)	25 (78.1%)	0.008
	Mild	466 (5.1%)	384 (4.6%)	82 (10.7%)		461 (5.1%)	5 (15.6%)	
	Moderate	126 (1.4%)	95 (1.1%)	31 (4.1%)		124 (1.4%)	2 (6.3%)	
	Severe	14 (0.2%)	9 (0.1%)	5 (0.7%)		14 (0.2%)	0 (0.0%)	
CNSI, n, %		623 (6.9%)	526 (6.3%)	97 (12.7%)	<0.001	622 (6.9%)	1 (3.1%)	0.723
ICH, n, %		49 (0.5%)	30 (0.4%)	19 (2.5%)	<0.001	47 (0.5%)	2 (6.2%)	<0.001
Timing of bleeding, n, %	Preoperative	3 (6.1%)	3 (10.0%)	0 (0.0%)	0.143	3 (6.4%)	0 (0.0%)	0.245
	Intraoperative	6 (12.2%)	2 (6.7%)	4 (21.1%)		5 (10.6%)	1 (50.0%)	
	Postoperative	40 (81.6%)	25 (83.3%)	15 (78.9%)		39 (83.0%)	1 (50.0%)	
Duration of bleeding, n, %	<6 h	24 (49.0%)	12 (50.0%)	9 (47.4%)	0.314	22 (46.8%)	2 (100.0%)	0.428
	6—24 h	6 (12.2%)	2 (6.7%)	4 (21.1%)		6 (12.8%)	0 (0.0%)	
	>24 h	16 (32.7%)	10 (33.3%)	6 (31.6%)		16 (34.0%)	0 (0.0%)	
DVT, n, %						743 (8.2%)	23 (71.9%)	<0.001
DHS, days, median(IQR)		12 (9–15)	12 (9–14)	15 (12–21)	<0.001	12 (9–15)	17.5 (14.5–22.5)	<0.001

COPD, chronic obstructive pulmonary disease; CHD, Coronary heart disease; CNSI, central nervous system infection; DVT, deep vein thrombosis; DHS, Duration of hospital stay; NS, nephrotic syndrome; OSAHS, obstructive sleep apnea hypopnea syndrome; PE, pulmonary thromboembolism; ICH, intracerebral hemorrhage; SLE, systemic lupus erythematosus.*Item distribution with statistical differences (*p* < 0.05) between the DVT group and PE group.

An analysis of the comorbidity characteristic data between the 8,301 non-SDVT participants and the 766 SDVT (Table 1) revealed statistically significant differences in terms of asthma (*p* < 0.001), atrial fibrillation (*p* = 0.047), heart failure (*p* < 0.001), coronary heart disease (CHD; *p* < 0.001), hypertension (*p* = 0.047), Hyperlipidemia (*p* < 0.001), varicose veins (*p* = 0.001), diabetes (*p* < 0.001), hypothyroidism (*p* < 0.001), hypoproteinemia (*p* < 0.001), and renal insufficiency (*p* < 0.001). Furthermore, analysis of comorbidity information between 9,035 non-PE and 32 SPE participants revealed statistically significant differences in heart failure (*p* = 0.014) and renal insufficiency (*p* = 0.011).

Meanwhile, An analysis of the complications characteristic data between the non-SDVT/non-PE participants and the SDVT/SPE (Table 1) revealed statistically significant differences in terms of pneumonia (*p* < 0.001), ICH (*p* < 0.001), anemia (*p* = 0.008). The incidence of pneumonia was 34.4% for SPE plus pneumonia higher than 17.4% for SDVT plus pneumonia (X^2^ = 6.01, *p* = 0.014). Among the 49 patients undergoing meningioma surgery, all were identified to have ICH without prior use of anticoagulant medications (0.5%), of whom 81.6% experienced the condition postoperatively, with 49% occurring within 6 h after surgery. The incidence of ICH was significantly high for both SDVT and SPE patients (both, *p* < 0.001): 2.5% for SDVT plus ICH and 6.2% for SPE plus ICH, surpassing the ICH incidence in the overall patient population (*p* < 0.001).

SDVT was present in 71.9% of SPE patients, which was a significantly higher proportion compared with that in the non-SPE population (8.2%). The mean duration of hospitalization was 13.12 ± 6.1 days. Both SDVT and SPE were associated with prolonged hospital stay (*p* < 0.001). Specifically, the average length of hospitalization was 17.72 ± 9.93 days for SDVT patients and 19.50 ± 9.48 days for SPE patients (Table 1).

### The univariate analyses for SDVT/SPE

**Figure 2:**
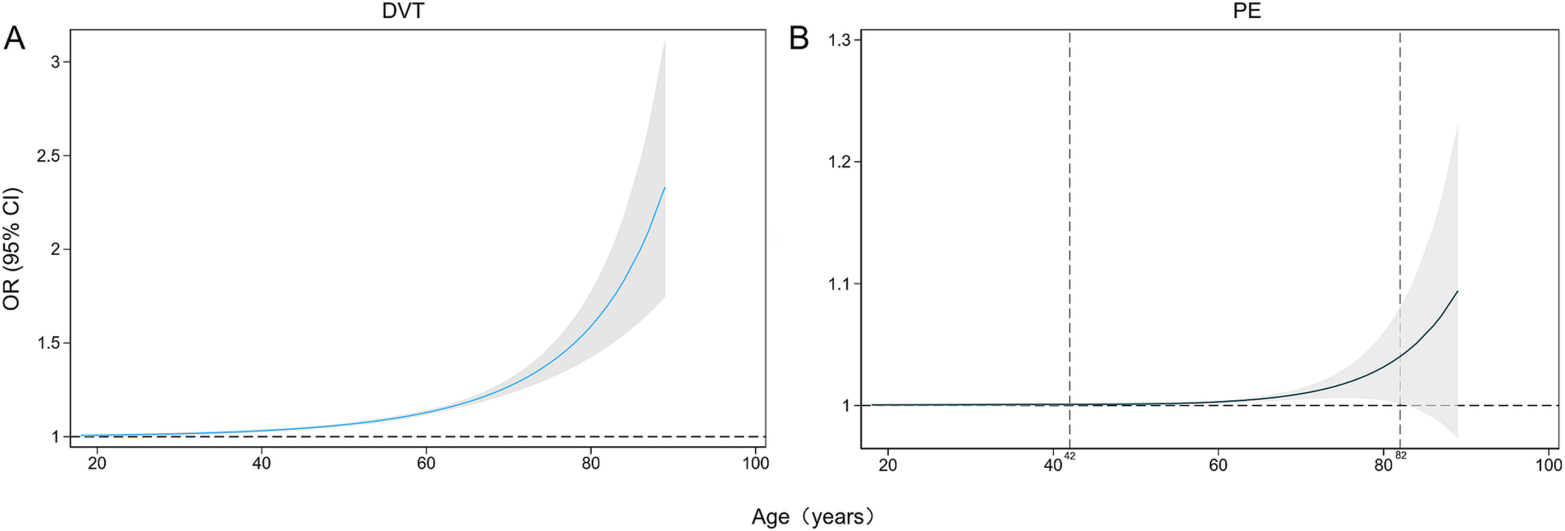
Restricted cubic splines of the impacts of age (years) on the incidences of DVT **(A)** and PE **(B)** as percentages (%). DVT, deep vein thrombosis; PE, pulmonary embolism.

Age emerged as a associated factor for the development of SDVT, with an odds ratio (OR) of 1.07 (95% confidence interval [CI] 1.06–1.08). Restricted cubic splines revealed a nonlinear, increasing trend in the incidence of SDVT with age for patients aged 18–89 years (Table 2; Figure 2A). Similarly, age was significantly associated with the development of SPE, with an OR of 1.09 (95% CI 1.05–1.13). Restricted cubic splines showed that the risk of SPE increased in patients aged 42–82 years, as follows: the OR for SPE increased from 1.001 (95% CI 1.000–1.002) at 42 years to 1.044 (95% CI 1.001–1.088) at 82 years (Table 2; Figure 2B). To facilitate the comparisons, univariate logistic regression applied to artificially constructed age group models indicated that model 3 had the optimal Akaike information criterion value, and the incidence of SPE in individuals aged ≥ 60 years was 4.58 times (95% CI 1.37–15.33) higher than that in individuals aged < 40 years. Subsequent comparative results, as presented in model 1, indicated that the incidence of SPE in individuals aged ≥ 80 years was 8.58 times (95% CI 1.02–72.03) higher than that of individuals aged 40–60 years (Table 3).

**Table 2:** Results of the univariate and multivariate analyses of age as a risk factor for DVT/PE.

Age	Logistic			Restricted cubic splines	
	OR	ROC	*p* value	Age group (years)	*p* value
DVT	1.07 (1.06–1.08)	0.69 (0.67–0.71)	<0.001	18–89	<0.001
PE	1.09 (1.05–1.13)	0.75 (0.67–0.84)	<0.001	42–82	<0.001

DVT, deep vein thrombosis; PE, pulmonary thrombosis.

**Table 3:** Results of the univariate logistic analysis of age in PE.

Age	Model 1	Model 2	Model 3	Model 4
	OR	OR	OR	OR
<40^#^	–	–	–	–
~45	–	–	–	
~50	0.34 (0.03–3.24)	0.21 (0.02–2.00)		
~55	0.29 (0.03–2.79)			
~60	1.48 (0.35–6.20)	0.88 (0.22–3.52)	0.62 (0.16–2.33)	
~65	4.17 (1.14–15.28)			1.21 (0.35–4.14)
~70	4.34 (1.12–16.83)	4.24 (1.24–14.48)		
~75	3.43 (0.57–20.63)			
~80	11.77 (1.93–71.47)	5.31 (1.18–23.85)		
~85	35.77 (3.49–366.95)*			
~90	–	27.35 (2.71–276.43)	4.58 (1.37–15.33)	5.00 (1.41–17.74)
AIC	405	404	403	418
BIC	468	447	424	439

# with the 0–40 years age group as the baseline. Model 1: Univariate analysis of PE in groups with age <40 as the baseline, with a 5-year age interval. Model 2: Univariate analysis of PE in groups with age <40 as the baseline, with a 10-year age interval. Model 3: Univariate analysis of PE in groups with age <40 as the baseline, for the 40–59 and 60–90 age groups. Model 4: Univariate analysis of PE in groups with age <40 as the baseline, for the 40–64 and 65–90 age groups. *Model 1: The odds ratio (OR) for the 80–85 years age group versus the 60–65 years age group was 8.58 [95% confidence interval (CI): 1.02–72.03], with a *p* value of 0.048. PE, pulmonary embolism; AIC, Akaike information criterion; BIC, Bayesian information criterion.

Logistic regression analysis revealed that comorbidity and complications were also significant associated factors for SDVT, such as respiratory system (asthma OR = 2.86, 95% CI 1.07–7.69), circulatory system (atrial fibrillation OR = 6.03, 95% CI 2.88–12.64; heart failure OR = 6.93, 95% CI 3.94–12.17; CHD OR = 2.04, 95% CI 1.06–3.89; Hypertension OR = 1.78, 95% CI 1.52–2.08; Varicose veins OR = 3.21, 95% CI 1.68–6.13), endocrine system (Hyperlipidemia OR = 2.17, 95% CI 1.53–3.06; Diabetes OR = 1.56, 95% CI 1.24–1.95; Hypothyroidism OR = 3.13, 95% CI 2.24–4.38), urinary system (Hypoproteinemia OR = 2.60, 95% CI 2.18–3.11; Renal insufficiency OR = 4.81, 95% CI 2.56–9.05), infections (Pneumonia OR = 5.85, 95% CI 4.69–7.29; CNSI OR = 2.14, 95% CI 1.70–2.70), ICH (OR = 7.01, 95% CI 3.93–12.52), and anemia. Among them, only a portion are associated factors for PE (heart failure OR = 11.98, 95% CI 2.79–51.49; Renal insufficiency OR = 13.62, 95% CI 3.16–58.76; Pneumonia OR = 11.02, 95% CI 5.28–23.01; ICH OR = 12.75, 95% CI 2.96–54.88; DVT OR = 28.52, 95% CI 13.15–61.86; and anemia; Table 4).

**Table 4:** Results of the univariate and multivariate logistic regression analyses of DVT and PE.

Factors	DVT				PE			
	Univariate	*p*	Multivariate	*p*	Univariate	*p*	Multivariate	*p*
Age	1.07 (1.06–1.08)	<0.001	1.06 (1.05–1.07)	<0.001	1.09 (1.05–1.13)	<0.001	1.05 (1.01–1.09)	0.009
Asthma	2.86 (1.07–7.69)	0.037						
Atrial fibrillation	6.03 (2.88–12.64)	<0.001	2.52 (1.12–5.58)	0.025				
Heart failure	6.93 (3.94–12.17)	<0.001	2.07 (1.09–3.95)	0.027	11.98 (2.79–51.49)	0.001		
CHD	2.04 (1.06–3.89)	<0.001						
Hypertension	1.78 (1.52–2.08)	<0.001			2.00 (0.98–4.11)	0.057		
Hyperlipidemia	2.17 (1.53–3.06)	<0.001	1.53 (1.06–2.22)	0.023				
Varicose veins	3.21 (1.68–6.13)	<0.001	2.53 (1.29–4.95)	0.007				
Diabetes	1.56 (1.24–1.95)	<0.001						
Hypothyroidism	3.13 (2.24–4.38)	<0.001	2.07 (1.42–3.01)	<0.001				
Hypoproteinemia	2.60 (2.18–3.11)	<0.001	1.73 (1.42–2.11)	<0.001				
Renal insufficiency	4.81 (2.56–9.05)	<0.001			13.62 (3.16–58.76)	<0.001		
Pneumonia anemia	5.85 (4.69–7.29)	<0.001	3.51 (2.75–4.48)	<0.001	11.02 (5.28–23.01)	<0.001	3.60 (1.65–7.85)	0.001
Mild	2.57 (2.00–3.31)	<0.001	2.05 (1.56–2.70)	<0.001	3.66 (1.39–9.60)	0.008		
Moderate	3.93 (2.60–5.95)	<0.001	2.72 (1.69–4.37)	<0.001	5.44 (1.28–23.23)	0.022		
Severe	6.70 (2.24–20.04)	0.001	3.16 (0.88–11.34)	0.077				
CNSI	2.14 (1.70–2.70)	<0.001	1.83 (1.42–2.35)	<0.001				
ICH	7.01 (3.93–12.52)	<0.001	5.79 (3.01–11.13)	<0.001	12.75 (2.96–54.88)	0.001		
DVT					28.52 (13.15–61.86)	<0.001	15.88 (6.94–36.35)	<0.001

CHD, Coronary heart disease; CNSI, central nervous system infection; DVT, deep vein thrombosis; PE, pulmonary thromboembolism; ICH, Intracerebral hemorrhage.

### The multivariate analyses for SDVT/SPE

**Figure 3:**
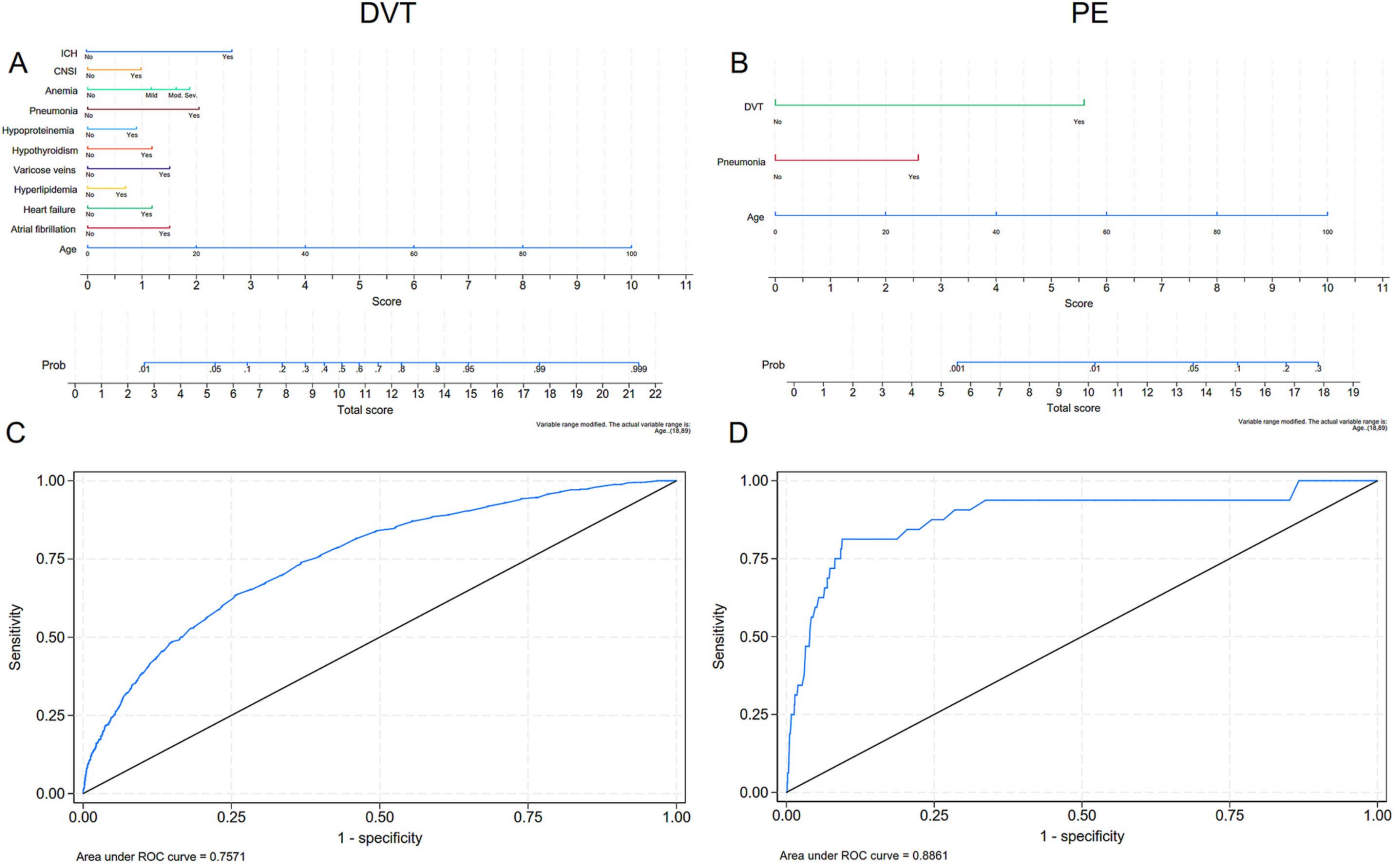
Multivariate modeling of the incidence rates of DVT and PE with nomograms and receiver operating characteristic (ROC) curves. **(A, C)** Nomograms and ROC curves of the incidence of DVT; **(B, D)** Nomograms and ROC curves of the incidence of PE. DVT, deep vein thrombosis; PE, pulmonary embolism.

Multivariate logistic regression analysis revealed that SDVT prediction models identified significant predictors, including age, six chronic diseases, ICH, and two infections, with an ROC curve area of 0.757. For predictive purposes, the nomogram shows that the top three are the cumulative age (OR: 1.06; 95% CI 1.05–1.07), pneumonia (OR: 3.51; 95% CI 2.75–4.48) and ICH (OR: 5.79; 95% CI 3.01–11.13) were significantly associated with the model, with the total score for the SDVT multi-factor model ranging from 1.8 to 24.2 points (probability 0.01–0.99). The SPE prediction model identified age, pneumonia, and DVT as significant predictors, with an ROC curve area of 0.886. For predictive purposes, the nomogram demonstrated that the cumulative age (OR: 1.05; 95% CI 1.01–1.09), pneumonia (OR: 3.60; 95% CI 1.65–7.85), and SDVT (OR: 15.88; 95% CI 6.94–36.35) were significantly associated with the model, with the total score for the SPE multi-factor model ranging from 1.8 to 17.1 points (probability 0.00–0.22; Table 4; Figure 3). ICH was included in the DVT model but excluded from the PE model due to collinearity (Kendall’s Tau-b = 0.08, *p* = 0.019, Vif = 1.01). This improved the model’s marginal effect, showing a 30% (95%CI 17–44%) increase in DVT incidence without significant impact on PE (*p* = 0.185; Supplementary Table 2; Supplementary Figure 1) Through the calibration curve, it was found that the consistency between the DVT/PE model training set and test set is good, but there is a certain overestimation of the incidence rate (Supplementary Figure 2).

## Discussion

In our retrospective analysis of data for 9,067 patients with meningiomas who underwent surgical procedures between January 2019 and June 2024, the incidence of SDVT was 8.4%, while the incidence of SPE was 0.4%. Univariate and multivariate analyses indicated that age, atrial fibrillation, heart failure, varicose veins, hyperlipidemia, hypothyroidism, hypoproteinemia, pneumonia, CNSI, ICH, and anemia were contributors to the development of SDVT, whereas age, pneumonia, and SDVT were identified as risk factors for SPE.

The incidence of SDVT following meningioma surgery at our center was 8.4%, similar to the incidence (8.7%) reported by Rizzo et al. (9), slightly higher than the 3–4% incidence commonly cited in recent studies (3, 4, 11), and significantly lower than that (72%) described by Sawaya et al. (5). These differences may be explained by advancements in DVT diagnostic technologies and an enhanced awareness of postoperative DVT (4). Historically, DVT was diagnosed using iodine 125-labeled fibrinogen imaging of the legs, which is less specific than current imaging methods, potentially leading to an overestimation of the incidence of postoperative DVT (5). Recently, ultrasonography has emerged as a simple and accessible method for diagnosing DVT, with high sensitivity and specificity (12). Meanwhile, with the increased emphasis on preventing DVT during the perioperative period in neurosurgery (3, 11) and the use of biomarkers such as D-dimer (13), earlier diagnosis of DVT has been achieved. The incidence of SPE was 0.4% in our study, which is lower than the incidence (1.7%) reported in the United States National Surgical Quality Improvement Program from 2006 to 2014 (10). This discrepancy can be attributed to the proactive assessment of preoperative DVT risk factors, intraoperative elevation of the lower limbs, postoperative monitoring for DVT, early postoperative mobilization, and active implementation of physical and pharmacological prophylactic measures. It is well recognized that the incidence of postoperative PE can be reduced by standardized regimens (14).

Age was identified as an independent risk factor for both SDVT and SPE in this study (1). A study from 1992 assessed the factors influencing mortality following meningioma surgery (15). The results indicated a lower mortality rate in patients aged < 45 years and a higher mortality rate in those aged ≥ 65 years, compared with patients in other age groups. However, the study preceded recent developments in the diagnosis and management of VTE and had a limited sample size. Another study of 275 meningioma cases in Italy indicated that age ≥ 60 years was a significant contributing factor in VTE development (4), and an analysis of a dataset of 5,036 meningioma cases from the United States National Surgical Quality Improvement Program demonstrated that age ≥ 65 years was an independent risk factor for VTE (8). The data in our study were obtained from a large number of patients from China and confirmed that age is an independent risk factor for both DVT and PE. Within the age range of 18–89 years, we observed a nonlinear increasing trend for the risk of SDVT. The risk of SPE increased by 4.58 times in individuals aged ≥ 60 years, and the risk for patients aged ≥ 80 years was 8.58 times higher compared with those in the 60–80 years group. Restricted cubic splines further revealed that the age range of 42–82 years was associated with a significant risk of SPE. Thus, for the Chinese population, in addition to the conventionally recognized high-risk age group of ≥ 60 years, the 42–60 years group also warrants increased attention. Regional SDVT/SPE distribution showed a significant correlation with age. At our institution in Northeast, North, and East China, individuals aged ≥65 years exhibited the highest meningioma prevalence. National population census data demonstrate a notably higher proportion of elderly residents in northern versus southern regions (16). This demographic pattern likely contributes to the observed higher SDVT/SPE rates in northern versus southern populations at our center; however, the study’s single-center design and population heterogeneity limited its ability to achieve statistical significance.

ICH is a significant risk factor for both DVT and PE (17). In patients with ICH, there is venous stasis due to immobility and hemiplegia, endothelial injury caused by invasive operations, and hypercoagulability as a result of dehydrating, hemostatic and antifibrinolytic agents (16). Postoperative venous VTE, including DVT and PE, occurs with notable frequency in patients who experience ICH (18). Studies show a 2–4% incidence of VTE following ICH, with DVT accounting for 1–2% and PE for 0.7–2% (19, 20). In a Chinese cohort of 314 patients with ICH, 5.7% developed DVT (21). In the present study, we found that the proportions of DVT and PE following ICH were 38.78 and 4.08%, respectively, which are higher rates than those reported in the literature. The reason is attributed to the routine ultrasound screening for DVT in postoperative ICH patients after transfer to the ICU, rather than relying on symptoms, has identified a higher incidence of DVT. Prospective studies report a 20–40% incidence of DVT detected via ultrasound during hospitalization (22–24). In postoperative ICH patients, elevated NIHSS scores (indicative of venous stasis due to paralysis severity and impaired consciousness) and elevated D-Dimer levels (23) (reflecting endothelial injury and hypercoagulability) contribute to VTE risk, consistent with Virchow’s triad (25).

Chronic comorbidities and infections were also identified as risk factor for both SDVT and SPE. Our study identified atrial fibrillation (26), heart failure (27), varicose veins (28), hypothyroidism (29), and pneumonia (30) as significant risk factors (OR ≥2, *p* < 0.005) for DVT. These findings align with international research indicating that cardiovascular disease leading to vascular stasis, hyperlipidemia resulting in a hypercoagulable state, and inflammation causing hypercoagulability, endothelial cell damage (14, 31) contributors to DVT risk. Notably, our predictive model, incorporating age, comorbidity, and complications, highlights the importance of early identification of at-risk patients and supports the need for tailored prophylactic measures. This multivariate model demonstrates promising predictive ability (ROC = 0.754), providing a practical tool for clinical decision-making. However, its overestimation of DVT risk also warrants our attention.

PE, like DVT, is influenced by a range of risk factors. Our study reveals that renal insufficiency, heart failure (9), pneumonia (30), ICH (32), and anemia (33) are significant risk factors for PE, which aligns with other studies linking these conditions to an increased risk of thromboembolism. Furthermore, DVT was strongly associated with PE (34), confirming the well-established connection between these two complications. The presence of DVT significantly increased the likelihood of PE, with an odds ratio of 28.52 (95%CI 13.15–61.86), emphasizing the need for early management of DVT to prevent subsequent PE. Notably, our predictive model excluded ICH due to multicollinearity, which incorporates age, pneumonia, and DVT, demonstrates promising predictive ability (ROC = 0.886), providing a practical tool for clinical decision-making. However, its overestimation of PE risk and limited prediction probability also warrants our attention.

Our study has several limitations. First, the dataset for this study included only hospitalized and symptomatic patients, which might have led to an underestimation of the incidence of postoperative DVT and PE (3, 11) compared with other studies that extended their data collection to 30 (4), 90 (9), or 120 (35, 36) days postoperatively. Second, all eligible reports that were included were retrospective. These retrospective studies, by definition, rely on imprecision and can suffer from data loss. Additionally, given the large volume of data, we selected specific demographic, comorbidity, and complications variables. Future research will aim to expand the dataset to include additional factors, such as blood type, body mass index, smoking status, homocysteine levels, tumor location and size, operative duration, surgical technique, postoperative management, and follow-up data.

## Conclusion

This article summarizes the findings of a comprehensive review of 9,067 patients with meningiomas undergoing surgical procedures between January 2019 and June 2024. The incidence of SDVT was 8.4%, while the incidence of SPE was 0.4%, reflecting differences in the assessment of complications following surgery. The study highlights significant associations between key risk factors and both SDVT and SPE, including age, atrial fibrillation, heart failure, varicose veins, hyperlipidemia, hypothyroidism, hypoproteinemia, pneumonia, CNSI, ICH, and anemia. Notably, in addition to the specific age threshold of > 60 years, a broader age range (42–82 years) is also a risk factor for postoperative SPE. Meanwhile, age, pneumonia, and DVT were identified as significant risk factors for SPE, with a multivariable predictive model (ROC = 0.886) demonstrating promising accuracy, offering a practical tool for clinical decision-making. However, the models overestimated DVT/PE risk and had limited predictive probability, necessitating further investigation. The study underscores the importance of multivariable analysis and early management in preventing complications. Future research should expand the dataset to include additional variables and refine the model for better clinical utility.

## Data Availability

The raw data supporting the conclusions of this article will be made available by the authors, without undue reservation.
