# Supplementary material for: Risk factors for postoperative thrombotic complications after meningioma resection: a retrospective single-center study in China
Source: Front Neurol. 2025 Jun 2;16:1579384. doi: 10.3389/fneur.2025.1579384 (PMC12171114; doi:10.3389/fneur.2025.1579384)
Supplement: Supplementary file 1 [file Table_1.docx]

Supplemental table 1. Regional and age distribution of surgical meningioma patients

|  | <40 years | 40-59 years | >=60 years | X^2^ | Test |
| --- | --- | --- | --- | --- | --- |
| total | 1,398 (15.41%) | 5,120 (56.51%) | 2,543 (28.08%) |  |  |
| Northeast | 532 (13.40%) | 2,106 (53.03%) | 1,333 (33.57%) | 189.55 | <0.001 |
| North China | 182 (11.83%) | 925 (60.14%) | 431 (28.02%) |  |  |
| Eastern China | 329 (17.43%) | 1,108 (58.69%) | 451 (23.89%) |  |  |
| Central South | 182 (20.40%) | 534 (59.87%) | 176 (19.73%) |  |  |
| Northwest | 80 (26.06%) | 172 (56.03%) | 55 (17.92%) |  |  |
| Southwest | 91 (19.74%) | 273 (59.22%) | 97 (21.04%) |  |  |
