# Supplementary material for: Risk factors for postoperative thrombotic complications after meningioma resection: a retrospective single-center study in China
Source: Front Neurol. 2025 Jun 2;16:1579384. doi: 10.3389/fneur.2025.1579384 (PMC12171114; doi:10.3389/fneur.2025.1579384)
Supplement: Supplementary file 2 [file Table_2.docx]

Supplemental table 2. Marginal effect of postoperative ICH on DVT/PE

| Model | Parameter | Margin | err. | 0.95% CI | | Z | P |
| --- | --- | --- | --- | --- | --- | --- | --- |
| DVT | Non-DVT | 0.920 | 0.003 | 0.910 | 0.920 | 314.000 | <0.001 |
|  | DVT | 0.080 | 0.003 | 0.080 | 0.090 | 29.020 | <0.001 |
|  | Non-DVT*Non-ICH | 0.920 | 0.003 | 0.910 | 0.920 | 316.000 | <0.001 |
|  | Non-DVT*ICH | 0.610 | 0.070 | 0.480 | 0.750 | 8.800 | <0.001 |
|  | DVT*Non-ICH | 0.080 | 0.003 | 0.080 | 0.090 | 28.540 | <0.001 |
|  | DVT*ICH | 0.390 | 0.070 | 0.250 | 0.520 | 5.570 | <0.001 |
|  | Non-DVT*(ICH - Non-ICH) | -0.300 | 0.070 | -0.440 | -0.170 | -4.380 | <0.001 |
|  | DVT*(ICH - Non-ICH) | 0.300 | 0.070 | 0.170 | 0.440 | 4.380 | <0.001 |
| PE | Non-PE | 0.996 | 0.001 | 0.995 | 0.998 | 1601.000 | <0.001 |
|  | PE | 0.080 | 0.003 | 0.080 | 0.090 | 29.020 | <0.001 |
|  | Non-PE*Non-ICH | 0.997 | 0.003 | 0.995 | 0.998 | 1643.000 | <0.001 |
|  | Non-PE*ICH | 0.959 | 0.028 | 0.904 | 1.015 | 33.930 | <0.001 |
|  | PE*Non-ICH | 0.003 | 0.001 | 0.002 | 0.005 | 5.490 | <0.001 |
|  | PE*ICH | 0.041 | 0.028 | -0.015 | 0.096 | 1.440 | <0.001 |
|  | Non-PE*(ICH - Non-ICH) | -0.037 | 0.028 | -0.093 | 0.018 | -1.330 | 0.185 |
|  | PE*(ICH - Non-ICH) | 0.037 | 0.028 | 0.093 | 0.018 | 1.330 | 0.185 |

DVT, deep vein thrombosis; PE, pulmonary thromboembolism; ICH, Intracerebral hemorrhage.
