# Supplementary figures and images for: Risk factors for postoperative thrombotic complications after meningioma resection: a retrospective single-center study in China
Source: Front Neurol. 2025 Jun 2;16:1579384. doi: 10.3389/fneur.2025.1579384 (PMC12171114; doi:10.3389/fneur.2025.1579384)

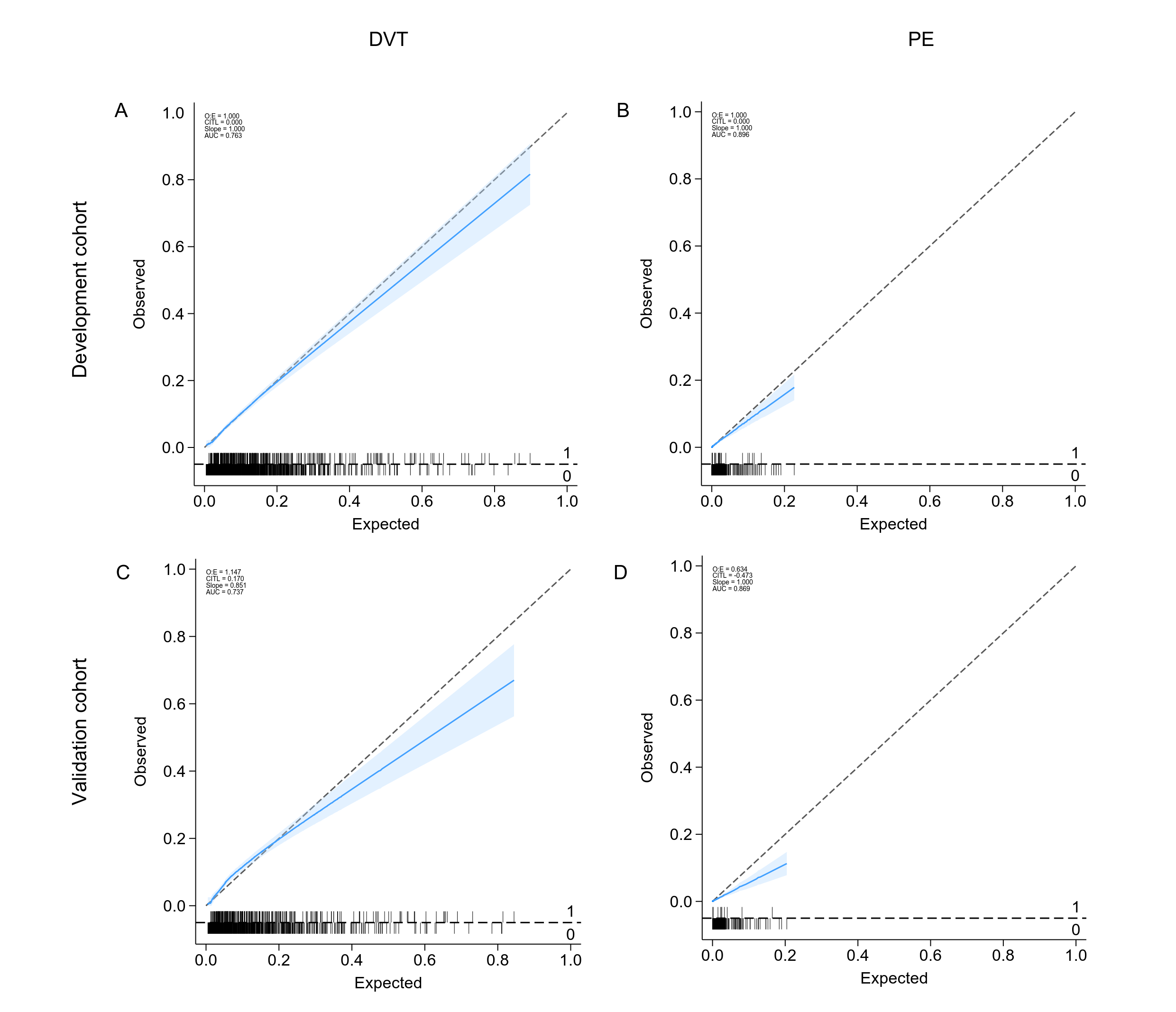

Supplement: Supplementary file 3 [file Image_1.tif]

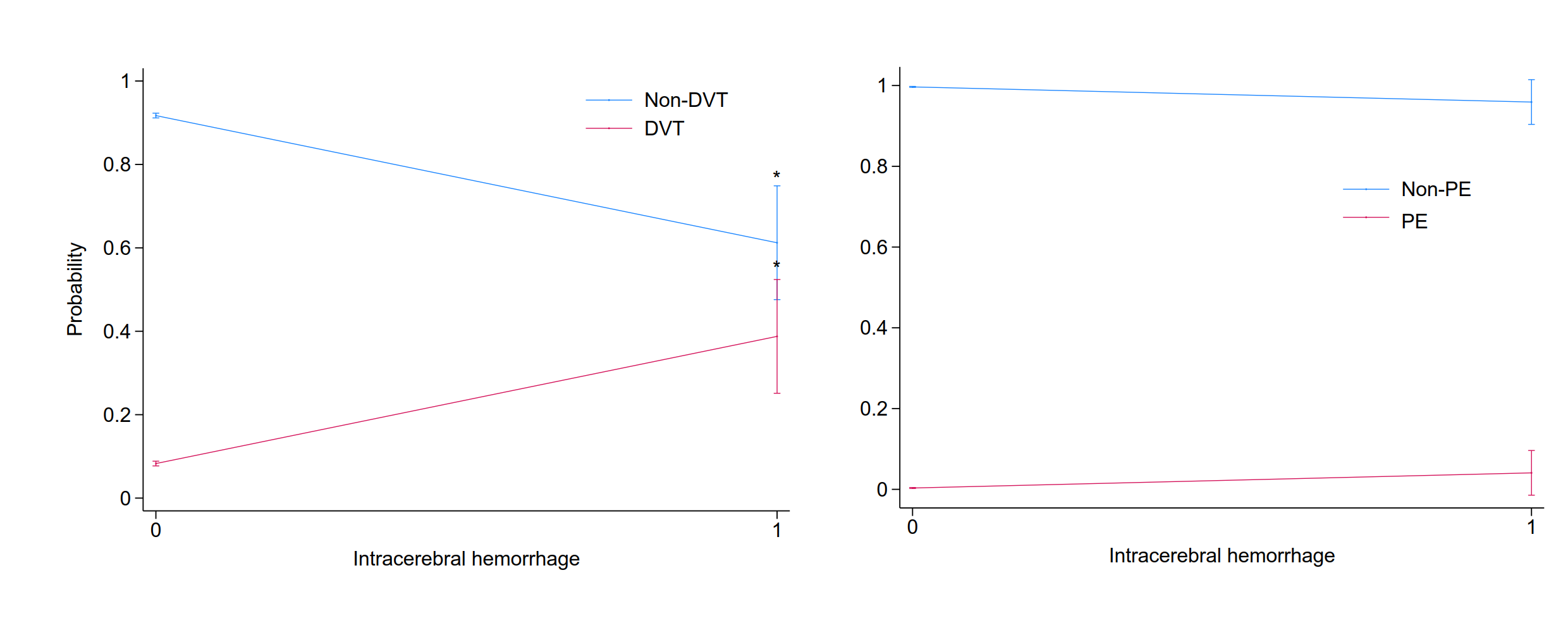

Supplement: Supplementary file 4 [file Image_2.tif]
